# Impressions

**Published:** 1856-12

**Authors:** H. R. Smith


### IMPRESSIONS.

BY H. R. SMITH, D. D. S., M. D.

In a former article I spoke of the preparation of the mouth for artificial teeth, and which, if strictly observed in detail, as I directed, will give us a good and solid foundation upon which we may place our artificial fixture of dental substitutes.

But, unless the foundation is properly prepared, it will be useless to proceed farther with the hope of making a satisfactory operation.

Next in importance to a good foundation, is a perfect impression of the part of the mouth, where we propose to apply our fixture. And I do not lay too much stress on my words when I say, that all future steps of our operation depend on getting a good impression. If the impression is bad, however well we may finish up the job, it will be unsatisfactory.

Too often are the pliers brought into requisition to remedy a defect arising from a bad impression. But let the young practitioner beware how he depends on that course to do his work, for even if he can force off work done in that way, in the end it will be far from profitable. If the job itself does not return, it prevents others from accumulating.

It is impossible to make a plate fit the mouth by bending with pliers, for if bent a little too much in any part, it will cause irritation by undue pressure. Therefore, we should depend on the pliers only as a last resort, not as a general rule.

Supposing the mouth to be in a sound and healthy condition, we should next proceed to the material for our impression. The substances generally used are white wax, yellow wax, and plaster, or gypsum. For a partial set I prefer wax, owing to the difficulty of removing plaster from the mouth. If the remaining teeth stand irregularly, or are smaller at the neck than at the crown, it would be impossible to remove plaster from the mouth without breaking the impression. And even if that could be done, the model could not be removed from the impression without breaking the teeth.

For this purpose, then, I prefer yellow wax, owing to its having a greater tenacity than white wax. The adhesive property is much injured in white wax by the bleaching process; and besides, very much of it is adulterated with stearine and tallow, so as to render it entirely unfit for dental purposes. It is also necessary to be very particular in selecting ellow wax, for very much of it has tallow mixed with it.

The tallow can generally be detected by color, taste, and smell. That having tallow has a dull whitish appearance, with fine, compact texture whon broken; while pure wax is not so close in texture, bright color, clear and crystalline.

To prepare wax for use, it should be melted and turned upon shallow tin dishes to the depth of one-fourth of an inch, or a little less, and left to cool. When cold, hold the dish over a spirit lamp to warm a little, when it can be removed with ease, and will be in the most convenient form for use.

If our wax is in this form, when our patient calls in, we can, in a very few moments, have it ready for our impression.

The wax should be quite soft, for unless it is, it will press too hard on the soft parts of the mouth, which will, by their elasticity, throw off the plates when adjusted, or by too much pressure on those parts, cause irritation.

In either case, our object will not be attained, that of a nicely fitting plate.

Next in importance is a proper impression cup. Many sizes should be at hand, and one selected that will fit and correspond with the contour of the alveola; for, if too wide or too narrow, a very poor impression will be the consequence. The cup being selected, the wax may now be warmed and placed in it—care being taken to have it smooth and even on the surface, without folds or creases, lest the surface of our impression, after taken, be uneven or crazed.

Before putting the wax into the patient's mouth, he should be directed to breathe through the nostrils, and to avoid swallowing, lest he strangle, and frustrate our object.

If the patient swallows during the time the wax is in the mouth, the wax coming in contact with the soft palate, will oftentimes make him sick, and we have to remove the wax before we perfect our work.

But if the patient will follow our directions about breathing and swallowing, very little difficulty will occur to hinder our getting a perfect impression.

For an upper impression, the operator should always stand on the right side of the patient, holding his cup in the right hand, the thumb and fore-finger grasping the handle, while the middle finger supports the middle of the cup beneath. In this manner, we have a firm hold, and one that gives us all the advantage of our work.

The patient's head being firmly supported against the chair, with the first finger of our left hand we gently extend the left corner of the mouth, while we first insert one corner of the cup of wax, turning it as it passes in, until we have it square and true in the mouth.

This part of the operation can not be performed with too much care, for in doing it we are liable to injure the corners of the mouth, and at the same time our patient will be very apt to notice our dexterity. Awkwardness at this stage of the operation is sure to be detected by the patient, and, to a certain degree, their confidence in our skill will be lessened. The lips now being raised out of the way of the wax, it is pressed up slowly and steadily as high as we wish, and while held firmly in this position, with the fore-finger of the left hand, the wax should be pressed up against the alveola and fossas in front. For in pressing up our cup, the wax is liable to roll out and leave a space, which is necessary to be filled by the plate.

During all of the operation up to this time, the firm hold of the cup should not be for a moment relaxed.

After waiting a minute or two for the wax to harden, it may be removed from the mouth. Loosen the wax by raising the lip and working it gently, and letting the air pass gently beneath the wax.

If too much force is used, the wax will be so disarranged that we shall not make a perfect fit of our plate.

After the wax is well loosened, drop it down in the mouth, turn it sideways, and remove it from the mouth with great care, in the same way that it was introduced, being careful that the lips do not press upon the sides of the impression, thereby changing it.

After the impression is removed, we should examine the mouth, and compare the impression with it, to ascertain whether it is correct. If the impression does not exactly agree with the mouth, we should repeat the operation, for the first will not answer.

As all our success depends on this operation, we should not be content unless we are certain that our impression is perfect.

The young practitioner generally supposes this to be a very simple and easy operation, and therefore places very little importance on it. But, when he has struck up his plate, and finds it does not fit the mouth, he does not know where his difficulty is; hence the resort to the pliers to remedy the defect.

It is not the tyro alone who has this difficulty, but also the more advanced operator (when we find one who is willing to admit that he ever has any difficulty.)

It is alone to those who have difficulties to contend with, and the inexperienced operator to whom I address myself, and for the benefit of such I would be explicit.

Much practice and patience is necessary, in order to be successful in this particular.

The impression of the inferior alveola may be obtained from the same position, or by turning the patient's head towards you, and bringing yourself directly facing him. In the latter position, you have greater control of the mouth, and can see when the cup is in its proper place, before pressure is made. When the wax is adjusted, place the first and second finger of each hand on the sides of the cup, and the two thumbs beneath the chin, and in this condition you will make the pressure.

By this position, you can have full power over the mouth, and make the pressure evenly on both sides of the mouth.

It is not necessary to make pressure with the finger on the sides, if you have a proper cup, as is done in the upper impression.

The same precautions should be used in removing the impression from the mouth as before directed.

Plaster of Paris, or gypsum, is not often used, excepting for full upper sets. When used, the same impression cups will be sufficient, by taking a piece of softened wax, and making a rim on the heel of the cup, to prevent the plaster escaping before it is hardened.

If plaster is used, the patient should be particularly cautioned about breathing through the mouth or swallowing, for if he does, he is almost sure to strangle, and retard the operation.

The plaster should be mixed with water a little thicker than we use for taking our model, put into the cup, and just as it begins to harden or crystallize, it should be placed in the mouth, and then treated the same as the wax, avoiding all motion until the plaster is fully set, which will be in from one to three minutes. None but the best article of plaster should be used for impressions, and that very fine.

There are some cases of full dentures when plaster is preferable to wax, namely, where the mouth is soft at the sides of the hard palate opposite to the molar teeth.

These places are often so soft and elastic, that when wax is pressed up, they are forced out of their natural position. Then, when we come to try in our plate, we find that it does not fit the hard palate.

The elasticity of the soft portions keeps the plate down, and no adhesion is the result. But if we use plaster quite soft, we get a good and accurate impression, with these soft places in their natural position.

Whether plaster or wax is used, two impressions, at least, should be taken: Firstly, if one is in any way injured, we have another to resort to; secondly, if we have two models by comparing we can see if there is any variation between them; and lastly, if the plate fits both of them alike, after it is stamped, the presumption is, that it will fit the mouth in the same manner.

If the plate fits the mouth perfectly at first, the chances of making a satisfactory job are all in our favor; but if it does not, then we can never succeed in doing what our patient would expect, or ourselves desire.

The young practitioner should be taught to perform all his operations carefully, but especially to begin aright, and lay his foundations substantially; then in time he will succeed.

It may be asked why I did not take up some new subject, rather than the old and familiar one of taking impressions. My reasons are these: These same familiar subjects are the ones that are neglected by writers, while subjects which have little else but theory, engross the attention of our best and most able writers. Teach the child the alphabet and first principles, and he will of himself afterwards learn all the higher and more refined sciences. Such is my only apology, if any is needed.

